# Disposal of Waste from Tattoo and Beauty Parlors in Poland: A Survey-Based Analysis on Epidemiological Safety

**DOI:** 10.3390/ijerph182312673

**Published:** 2021-12-01

**Authors:** Anita Gębska-Kuczerowska, Izabela Kucharska, Agnieszka Segiet-Święcicka, Marcin Kuczerowski, Robert Gajda

**Affiliations:** 1 Collegium Medicum, Cardinal Stefan Wyszyński University, Kazimierza Wóycickiego 1/3, 01-938 Warsaw, Poland; 2 Chief Sanitary Inspectorate, Targowa 65, 03-729 Warsaw, Poland; i.kucharska@gis.gov.pl; 3 Faculty and Department of Experimental Physiology, Medical University of Warsaw, Żwirki i Wigury 61, 02-091 Warsaw, Poland; agnieszkasegiet@gmail.com; 4 Clinical Department of Oncological Gynecology and Obstetrics, Professor Witold Orlowski Independent Public Clinical Hospital, Czerniakowska 231, 00-416 Warsaw, Poland; mkuczerowski@vp.pl; 5 Gajda-Med Medical Center, ul. Piotra Skargi 23/29, 06-100 Pułtusk, Poland; gajda@gajdamed.pl

**Keywords:** professional infection risk, needle-stick injury, disposal of hazardous waste, tattoo parlors, beauty parlors

## Abstract

Appropriate waste management is increasingly relevant due to environmental and infectious disease transmission concerns. An anonymous observational cross-sectional study was conducted from 2013–2017 of 262 tattooists and 824 beauticians throughout Poland. Knowledge, attitudes, behavior, and compliance with blood-borne infection controls and correct waste disposal were assessed. Tattooists correctly addressed hazardous waste significantly more often than did beauticians (83.3% vs. 44.8%). Medical waste was collected by a specialist company in 90.1% of tattoo parlors and 63.3%of beauty parlors. Tattooists correctly used and disposed of sharps more frequently than beauticians (93.1% vs. 68.9%); however, 46.4% of beauticians and 12.4% of tattooists discarded waste into municipal trash, including sharps (27.1% and 2.6%, respectively). Incorrect collection and labeling of biological waste present occupational risk to waste disposal personnel. Education and instructional controls could improve health safety in this industry. Biological waste management processes are restrictive for medical services and liberal for beauty services, an industry for which they should also be applied more comprehensively.

## 1. Introduction

Many countries in the European Union have introduced restrictions regarding the conditions under which tattoo and beauty parlors may perform their services, the purpose of which is to ensure that the services provided are safe [1,2].

Many extant studies have considered hygiene as a potential risk factor for transmission of infectious diseases [3] and the need to update professional knowledge regarding this subject [4]. Attention is increasingly being paid to issues surrounding the health risks associated with the performance of services together with the materials and tools that are used [5,6,7]. For understandable reasons, health risk assessments in the beauty services industry are considered both in relation to the transmission of infectious diseases and the health consequences that may arise in the future [8,9].

The growing interest in beauty services, which is associated with a higher risk of complications, has resulted in the need for the amendment of legal regulations in the area of health safety for risk-bearing services (RES AP 2008 (1) and further amendments) [10]. It appears that the issue of waste/garbage disposal from tattoo and beauty parlors has been marginalized.

From the public health perspective, the important topic of waste disposal from non-medical services has received minimal interest, with more attention being paid to the disposal of waste from healthcare facilities and emphasis being placed on ecological aspects [11,12].

The results of our study of blood-borne infections (BBI) indicate the need to consider the problem from a different perspective due to the increased interest in, and invasiveness of, procedures in non-medical services [13,14]. Not only is the risk for the customer increasing, but also arethe health and environmental risks due to the production of biologically hazardous waste, similar to the medical services industry.The aim of this study was to assess the health risks associated with waste disposal from tattoo and beauty salons in Poland.

## 2. Methods

Observational research was conducted in each of Poland’s 16 provinces in tattoo and beauty parlors. The selection of tattoo and beauty parlors was quasi-random and based on a register of service sites (due to incomplete data in the provinces’ registries on operating beauty salons and tattoo parlors). Questionnaires were collected in all provinces by the State Sanitary Inspectorate, a branch of the Administration and Legal Authority. The research results and database collected by the State Sanitary Inspectorate were used to support the country’s BBI education program [15]. The questionnaires for both professional groups, namely tattoo and beauty parlors, were developed together with the State Sanitary Inspectorate (local governmental administration), after consultation with professional groups and specialists in infectious diseases and epidemiology. Audits and data collection (100% response rate) were performed between 2013 and 2015. Demographic characteristics of respondents’ group is presented in Table 1. All discrepancies in questionnaire answers were explained simultaneously (ad hoc and updated during data quality verification until 2015/17). The questionnaires were divided into sections relating to the staff and the work site. Respondents’ participation in the study was voluntary and anonymous, and informed consent was obtained before interviews were conducted. The study proposal and design were accepted by the “Project HCV” Steering Committee and were undertaken at tattoo and beauty parlors by State Sanitary Inspectorate staff during their routine duties.

### 2.1. Statistical Analysis

We analyzed the risk factors for BBI associated with waste disposal practices of tattooists and beauticians. Statistical analysis was performed on data collected from 824 people performing beauty services and 262 people performing tattoo services. No data imputation methods were used to complete missing data. Data were analyzed using descriptive methods and bivariate analyses. In the case of continuous variables, the number of non-empty observations, mean, standard deviation, minimum, and maximum were reported. For categorical variables, the number and percentage of occurrences were reported. To compare the distribution of continuous variables between the two subgroups, first the normality of the distribution of the variable in these subgroups was tested using the Shapiro–Wilk test, then in the case of a variables with normal distribution, Student’s *t*-test was used to test differences between the subgroups; otherwise, the Mann–Whitney test was used. In the case of categorical variables, Fisher’s test or chi-square test was used to test the differences in the distribution of variables between subgroups or to test the significance of the relationship between two categorical variables, depending on the expected size of each category. Two-sided tests were used in all analyses and a level of significance was set at 0.05. The statistical analysis was carried out using the R, version 3.4.0 (R Core Team, Vienna, Austria).

### 2.2. Role of the Funding Source

The study did not receive any external funding. The database was created for the Education Project on BBI and partially supported by the Swiss Contribution and Polish Ministry of Health (KIK35). The founders of the database had no role in the study design, data collection, data analysis, data interpretation, writing of the report, or the decision to publish. All authors had full access to all of the data in the study and had final responsibility for the decision to submit for publication.

## 3. Results and Discussion

### 3.1. Handling Waste in Tattoo and Beauty Parlors

In our study, tattooists were significantly more likely to correctly dispose of hazardous waste than persons performing cosmetic services(83.3% vs. 44.8%). In 90.1% of tattoo parlors, medical waste was collected by a specialized company, while this was only carried out in 63.3% of cosmetics parlors. Tattooists segregated waste and disposed of it in containers with other recyclable materials more often than beauticians (97.0% vs. 88.3%). At the same time, a much higher percentage of beauticians than tattooists threw waste into municipal trash (46.4% vs. 12.4%) or less used waste utilization methods by the burning/buried (1.6% vs. 0.4%; Table 2).

In the beauty services industry, with the invasive procedures and the use of sharp tools for this purpose, 53.3% of employees declared that proper waste disposal was carried out. This is slightly less than the results of a World Health Organization (WHO) and a United Nations Children’s Fund (UNICEF) study in the medical services sectorconducted in 2015 [16]. The joint international research initiative in 24 countries showed that only 58% of healthcare facilities met the conditions for safe waste disposal.

Among the professional group of beauticians, in the subgroup of people who did not participate in courses for beauticians, percentage of people who incorrectly disposed of wastewas higher than in the subgroup of people who participated in courses for beauticians (Table 3). Similarly, respondents from tattoo parlors, who had declared that they read medical textbooks, chose proper waste disposal significantly more often (Table 3).Thus, the results of our study confirm the general principle that experience, improving knowledge, and updating it are very important steps towards minimizing health risks, regardless of profession [17,18,19]. For tattoo parlors, the age of employees did not affect the choice of the form of waste disposal, while for beauty parlors, the choice of a safe form of waste disposal was related to the length of job experience (62.5% in the subgroup with job experience of 0–1 year, 69.0% for 2–5 years, 68.7% for 6–10 years, 80.6% for 11–20 years, and 80.0% for more than 20 years; *p* = 0.007).

Since the inspections by the State Sanitary Inspectorate, apart from the audit, were often of an instructional nature, the correct choices of service workers observed in the results of this study resulted more from positive reinforcement and additional knowledge than from the imposition of sanctions. This is consistent with the opinion of other researchers, that better long-term effects are achieved by understanding irregularities(discrepancies) and introducing changes on one’s own, thereby avoiding punishment [20,21,22].

### 3.2. Handling Sharp Waste in Tattoo Beauty Parlors

It is necessary to pre-segregate waste, label it, and select an appropriate disposal method, not only for environmental reasons but epidemiological safety. It is particularly important to dispose of sharp objects used during treatments due to the need to protect people from unintentional stabbing/injury [23]. This applies to employees who provide services with contaminated sharp tools, andto employees who pick up poorly protected sharp objects or incorrectly sorted and unmarked waste [23,24].

The current rules for the handling of sharp tools and contaminated instruments in non-medical services, from a logical point of view, should be as restrictive as those used in medical services [25,26,27,28,29]. However, the same views are not held in every country [26]. This can be explained by the different levels of automation of waste disposal and the lower risk for humans associated with increased levels of automation.

In many countries, the logistics of waste disposal are standardized by variouslaws, at different administrative levels, and by different authorities. For example, waste disposal in the USA is regulated by the Centers for Disease Control (CDC), Occupational Safety and Health Administration (OSHA), U.S. Food and Drug Administration(FDA), federal law, and local state law [30]. Many guide/recommendation documents contain valuable tips on the safety of all stages of waste disposal from storage, labeling, and receipt in order to minimize the health risk for people who must handle contaminated/hazardous materials [31].

In our study, most beauticians and tattooists did not disinfect/sterilize needles, and instead threw them directly into the trash (Table 4). Of statistical significance, more tattooists than beauticians secured sharp needles, put them into sharps containers, and gave them to a company that disposes of medical waste (97.4% vs. 72.9%; *p* < 0.001).At the same time, tattooists chose the safest forms of sharp waste disposal among all the listed forms (93.1% vs. 68.9%; *p* < 0.001).

However, employees of beauty parlors declared more often that they discarded waste into the municipal trash, and as many as 27.1% of beauticians and 2.6% of tattooists threw their sharp waste into the municipal trash, either directly or in a sharps container. Beauticians also disposed of their waste as medical waste much less often and used outsourcing contracts for sterilization less frequently (Table 5).

Considering the information about injuries experienced by beauty parlor employees caused by disposable one-time usage sharp equipment, such as needles, pre-filled syringes, or blades, and the information about the preferred forms of disposal, it should be assumed that these sharp objects are treated as municipal waste (not medical waste). Nienhaus concluded through a 22-year analysis that, in Germany, a decrease in the incidence of BBI among healthcare workers was caused by awareness of the risk and use of microbiologically safe devices [32]. Paradoxically, an increase from 30% to 50% was observed in the registered events of injury by needles for subcutaneous injections [32,33]. At the same time, people working in tattoo parlors did not report a large number of stabbing incidents with sharp objects as they were more likely to use a safe form of waste disposal, that is, treating waste as medical waste (Table 6).

According to WHO estimates, nearly 16 billion injections are administered worldwide every year, but not all syringes and needles are disposed of safely. Another problem from a public health perspective is the failure to recognize the issue of waste disposal in the dynamically developing non-medical services sector. The classification of waste is divided into infectious waste (contaminated), pathological waste, sharp waste (e.g., syringes, needles, disposable scalpels, and blades), chemical waste, pharmaceutical waste, cytotoxic or radioactive waste, and non-hazardous waste (general waste) [34]. Contemporary cosmetic and tattoo services produce the afore mentioned waste with the exception of cytotoxic, radioactive, and pathological waste. In the global view of WHO experts as to what constitutes sources of (medical) waste, the following were listed: hospitals and other health facilities, laboratories and research centers, mortuary and autopsy centers, animal research and testing laboratories, blood banks and collection services, andnursing homes for the elderly. Unfortunately, tattoo and beauty parlors were not included in these sources. In the modern world, beauty services have become more invasive and expansive, making them similar to medical services. Thus, it should be assumed that they may constitute an additional source of medical waste. Similar to the treatments themselves, contaminated waste can provide an additional source and route of transmission for BBI [35].

The weakness of this study is the lack of risk assessment from the direct waste quantity analysis, and only subjective assessments made by tattoo/beauty parlor employees. As WHO experts point out, in high-income countries, 0.5 kg of hazardous waste is generated per hospital bed per day, while the value is 0.2 kg in low-income countries [34].

Our research approach as well as the marginalization of the waste disposal problem, may result in an underestimation of the scale of the studied phenomenon. At the same time, this limits the possibility of assessing the global risk scale arising from incorrect waste disposal in the medical and non-medical service sectors.

Numerous studies showing the extent of various unresolved problems in the healthcare sector in this area, as well as new unresolved problems, indicate that the topic of medical waste disposal is a problem that urgently requires solutions [36,37,38]. A certain limitation of the survey may be that it was closed in 2016/17 under different circumstances of epidemic risk. However, although the study was closed with 2017 (updated through data curation), there have been no new changes to the topic since then, other than those resulting from new client contact procedures as a result of the COVID-19 pandemic. Another limitation was the completeness of some data; the percentage of missing data was rather high at up to 15% percent. We cannot exclude that data is missing not at random, there is a non-negligible risk that the respondents did not answer the questions in which they had not known the answer or had known that their behavior was incorrect, and hence the percentage of respondents with unsatisfactory knowledge or presenting dangerous behavior may be underestimated. Due to the above observation, the results of the study should be interpreted with greater caution, as the risk associated with tattooing and beautification may be underestimated (higher). A strength of this study is that its results could be practically implemented as waste policies with appropriate regulations and interventions if the issues are currently addressed in such regulations.

The current experience in the fight against the spread of infections, due to the COVID-19 pandemic, should sensitize many decision-makers to the fact that success in the fight against many pathogensmostly dependson a prevention approach [39]. According to the authors of the article, not only urgent action but also research is needed in this area. Similarly, Vinti et al. indicated in a recent systematic review based on the PRISMA statement that the problem of waste disposal, in terms of public health safety, requires further urgent and detailed research due to gaps in risk information [40].

## 4. Conclusions

Proper disposal of waste from non-medical services is an underappreciated and underestimated problem. Incorrect collection and lack of labeling and segregation, especially of sharp and biologically contaminated objects, is a potential occupational risk for many professionals participating invarious stages of the logistics chain of waste generation and disposal. Vocational education of at-risk groupsas well as general population education, would be effective ways to reduce the risk of transmission of infections and to introduce positive changes. Inspections by the State Sanitary Inspectorate and simultaneous education bodies are recommended. Job experience plays an important role in the correct/safe disposal of waste by professional beauticians. Among professional tattooists, this trend was not noted because they showed better pre-existing practical knowledge in this field.

## Figures and Tables

**Table 1:** Demographic characteristics of respondents (*n* = 1086).

Variable	Category	Tattooist *n* = 262(%)	Beautician *n* = 824(%)	*p*-Value
Gender	Female	61 (25.0)	804 (99.4)	<0.001
	Male	183 (75.0)	5(0.06)	
Education	Primary	4 (1.6)	1 (0.1)	<0.001
	Vocational	43 (17.7)	18 (2.2)	
	Post-secondary	22 (9.1)	271 (33.6)	
	Secondary	125 (51.4)	173 (21.5)	
	Bachelor’s degree	22 (9.1)	184 (22.8)	
	Master’s degree	27 (11.1)	159 (19.7)	
Age	Below 18 yrs old	1 (0.4)	3 (0.4)	0.002
	18–25	20 (8.2)	65 (8.0)	
	26–35	114 (46.5)	384 (47.3)	
	36–45	97 (39.6)	241 (29.7)	
	46–55	10 (4.1)	94 (11.6)	
	56+	3 (1.2)	25 (3.1)	
Work experience (Yrs)	*n*	241	792	0.446
	Mean	9.5	10.5	
	SD	6.0	7.9	
	Min	0.5	0.5	
	Max	30.0	44.0	

*n*—number of non-empty observations, SD—standard deviation, Min—minimum, Max—maximum.

**Table 2:** Disposal of waste by beauticians and tattooists.

Waste Is:	Category	Total *n* (%)	Beautician *n* (%)	Tattooist *n* (%)	*p*-Value
1. Thrown in the municipal trash	Yes	406 (38.8)	377 (46.4)	29 (12.4)	<0.001
	No	640 (61.2)	436 (53.6)	204 (87.6)	
2. Sorted and thrown into containers with other recyclable materials	Yes	102 (9.8)	95 (11.7)	7 (3.0)	<0.001
	No	944 (90.2)	718 (88.3)	226 (97.0)	
3. Collected by a medical waste company	Yes	725 (69.3)	515 (63.3)	210 (90.1)	<0.001
	No	321 (30.7)	298 (36.7)	23 (9.9)	
4. Burnt or buried	Yes	14 (1.3)	13 (1.6)	1 (0.4)	0.327
	No	1032 (98.7)	800 (98.4)	232 (99.6)	
5. Only correct answers by total (1-No, 2-No, 3-Yes, 4-No)	Yes	558 (53.3)	364 (44.8)	194 (83.3)	<0.001
	No	488 (46.7)	449 (55.2)	39 (16.7)	

**Table 3:** Disposal of waste and sources of knowledge of beauticians and tattooists.

Beauticians				
		Correct Disposal of Sharp Garbage * *n* (%)	Incorrect Disposal of Sharp Garbage *n* (%)	*p*-Value
Parlors are controlled by the State Sanitary Inspection	No	10 (62.5)	6 (37.5)	0.003
	Don’t know	8 (72.7)	3 (27.3)	
	Yes	545 (88.8)	69 (11.2)	
		Correct disposal of sharp garbage * *n* (%)	Incorrect disposal of sharp garbage *n* (%)	0.014
Participates in courses for beauticians	No	84 (80.0)	21 (20.0)	
	Yes	503 (89.2)	61 (10.8)	
		Correct disposal of garbage * *n* (%)	Incorrect disposal of garbage *n* (%)	0.036
Participates in courses for beauticians	No	72 (55.4)	58 (44.6)	
	Yes	439 (65.5)	231 (34.5)	
**Tattooists**				
		Correct disposal of garbage * *n* (%)	**Incorrect disposal of** **garbage *n* (%)**	0.038
Reads medical textbooks	No	79 (84.9)	14 (15.1)	
	Yes	127 (94.1)	8 (5.9)	

* In a non-piercing container and handed over to a medical waste disposal company.

**Table 4:** Handling of used needles by beauticians and tattooists.

Used Needles Are:	Category	Total *n* (%)	Beauticians *n* (%)	Tattooists *n* (%)	*p*-Value
1. Disinfected	Yes	37 (3.5)	32 (3.9)	5 (2.1)	0.231
	No	1009 (96.5)	781 (96.1)	228 (97.9)	
2. Sterilized	Yes	7 (0.7)	6 (0.7)	1 (0.4)	>0.999
	No	1039 (99.3)	807 (99.3)	232 (99.6)	
3. Put into a non-piercing container and then thrown in the municipal trash	Yes	63 (6.0)	54 (6.6)	9 (3.9)	0.158
	No	983 (94.0)	759 (93.4)	224 (96.1)	
4. Thrown directly into the municipal trash	Yes	14 (1.3)	12 (1.5)	2 (0.9)	0.747
	No	1032 (98.7)	801 (98.5)	231 (99.1)	
5. Put in a non-piercing container and handed over to a medical waste disposal company	Yes	820 (78.4)	593 (72.9)	227 (97.4)	<0.001
	No	226 (21.6)	220 (27.1)	6 (2.6)	
Total-correct all answers (1-no,2-no, 3-no,4-no, 5-yes)	Yes	777 (74.3)	560 (68.9)	217 (93.1)	<0.001
	No	269 (25.7)	253 (31.1)	16 (6.9)	

**Table 5:** Procedures using sharp tools and the choice of the correct form of disposal by beauty parlors.

Variable	Category	Put in a Non-Piercing Container and Handed over to a Medical Waste Disposal Company *n* (%)		*p*-Value
		No	Yes	
Garbage is thrown into the municipal trash	No	18 (22.2)	374 (64.2)	<0.001
	Yes	63 (77.8)	209 (35.8)	
Instruments are sterilized using internal resources	No	48 (59.3)	252 (44.3)	0.012
	Yes	33 (40.7)	317 (55.7)	

**Table 6:** Needle stick injury events and sharp waste disposal in tattoo parlors.

Variable	Category	Stabbing *n* (%)		*p*-Value
		No	Yes	
Put in a non-piercing container and handed over to a medical waste disposal company	No	2 (1.1)	3 (6.8)	0.048
	Yes	187 (98.9)	41 (93.2)	

## Data Availability

The corresponding author had full access to all anonymized data in the study and had the final responsibility regarding the decision to submit for publication. All authors have had access to the data. Additional access to the data can be provided upon reasonable request.
